# Mutations in *WDR62 *gene in Pakistani families with autosomal recessive primary microcephaly

**DOI:** 10.1186/1471-2377-11-119

**Published:** 2011-10-01

**Authors:** Rizwana Kousar, Muhammad Jawad Hassan, Bushra Khan, Sulman Basit, Saqib Mahmood, Asif Mir, Wasim Ahmad, Muhammad Ansar

**Affiliations:** 1 Department of Biochemistry, Faculty of Biological Sciences, Quaid-i-Azam University, Islamabad, Pakistan; 2 Department of Biochemistry, University of Health Sciences, Khayaban-e-Jamia Punjab, Lahore, 54600, Pakistan; 3 Department of Human Genetics and Molecular Biology, University of Health Sciences, Khayaban-e-Jamia Punjab, Lahore, 54600, Pakistan; 4 Department of Bioinformatics and Biotechnology, International Islamic university, H-10, Islamabad, Pakistan

## Abstract

**Background:**

Autosomal recessive primary microcephaly is a disorder of neurogenic mitosis that causes reduction in brain size. It is a rare heterogeneous condition with seven causative genes reported to date. Mutations in *WD repeat protein 62 *are associated with autosomal recessive primary microcephaly with cortical malformations. This study was initiated to screen *WDR62 *mutations in four consanguineous Pakistani families with autosomal recessive primary microcephaly.

**Methods:**

As part of a large study to detect the genetic basis of primary microcephaly in Pakistan, homozygosity mapping and DNA sequencing was used to explore the genetic basis of autosomal recessive primary microcephaly in four families.

**Results:**

Four out of 100 families recruited in the study revealed linkage to the *MCPH2 *locus on chromosome 19, which harbor *WDR62 *gene. DNA sequencing in these *MCPH2 *linked families result in the identification of a novel nonsense mutation (p.Q648X) and three previously known mutations.

**Conclusion:**

Our data indicate that *WDR62 *mutations cause about 4% of autosomal recessive primary microcephaly in Pakistan.

## Background

Primary autosomal recessive microcephaly (MCPH) is a congenital disorder, initially characterized by architecturally normal brain but of reduced size associated with mild to moderate mental retardation. However recent studies have broadened the phenotypic spectrum of MCPH gene mutations by associating additional cortical anomalies [1-4]. Autosomal recessive primary microcephaly is a heterogeneous disorder with seven causative genes reported to date. These include *MICROCEPHALIN *at *MCPH1 *[5], *CDK5RAP2 *at *MCPH3 *[6]*CEP152 *at *MCPH4 *[7], *ASPM *at *MCPH5 *[8], *CENPJ *at *MCPH6 *[6], *STIL *at *MCPH7 *[9] and *WDR62 *at *MCPH2 *[1-3].

In Pakistani population, mutations in *WDR62 *are second most common cause of MCPH. *WDR62 *(NM_001083961) gene maps to chromosome 19q13.12 and encodes two alternative *WDR62 *transcripts in humans, however full length transcripts comprise 32 coding exons encoding a 1,523 amino acid protein. Mutations in *WDR62 *in five Pakistani families have been reported by *Nicholas et al*. [2]. In present work, we report identification of a novel and three known mutation in *WDR62 *gene in four additional Pakistani families with autosomal recessive primary microcephaly.

## Methods

### Study subjects

Approval to conduct present study was obtained from the Institutional Review Board of Quaid-i-Azam University (Quaid-I-Azam U IRB#1- Biomedical; IORG0002926; IRB00003532), Islamabad. Written informed consents were taken from the parents of affected individuals who agreed to participate in the current study by conforming to Helsinki Declaration and local legislations. As part of a large study to address the prevalence and genetic basis of primary microcephaly in Pakistan, 100 families were initially identified and recruited based on the reduced head circumference of affected individuals assessed during field visits in different areas of the Pakistan. Affected probands of cooperative families were clinically examined at Children Hospital, Lahore to record relevant biometric data followed by radiological examination (X-rays and CT scan).

### Extraction of Genomic DNA and Genotyping

Genomic DNA was extracted from venous blood samples following a standard phenol chloroform procedure. Homozygosity mapping was performed in these families, to test linkage to *MCPH1-6 *loci as described previously in [10] and to *MCPH7 *by genotyping D1S2797, D1S3714, D1S2720, D1S3315, D1S2748, D1S1661 and D1S386 markers. Genetic map distances of the markers were obtained from the Rutgers combined linkage-physical map (36.2) of the human genome [11].

### *WDR62 *sequencing

*WDR62 *gene was sequenced in one obligate carrier and two affected members from each of the four families linked to the *MCPH2 *locus by PCR, amplifying all 32 coding exons and splice junction sites by using a set of 27 primer pairs. Purification of the PCR-amplified DNA was performed with commercially available kit (Marligen Biosciences, Ijamsville, Maryland) and sequencing was performed with Big Dye Terminator v3.1 Cycle Sequencing Kit, with an ABI Prism 310 Genetic Analyzer (Applera, Foster City, California). Sequence variants were identified via Bioedit sequence alignment editor version 6.0.7)

## Results

**Figure 1:**
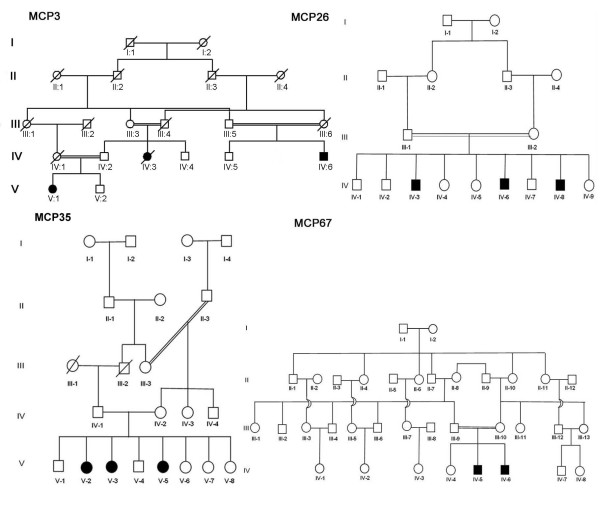
**Pedigrees of families MCP3, MCP26, MCP35 and MCP67 with *WDR62 *mutations**. Clear symbols represent unaffected individuals, Filled squares represent affected males. Double lines between symbols are representatives of consanguineous unions.

**Figure 2:**
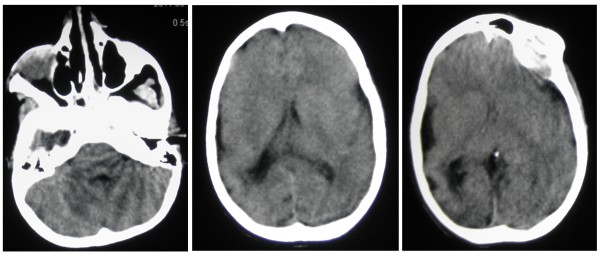
**The computerized tomographic (CT) scan of affected individual from family MCP67**. The axial CT scan depicts ill defined gryal and nuclei pattern in the affected individual (IV-5) of family MCP67 with homozygous nonsense *WDR62 *mutation.

By homozygosity mapping, four families resulted in linkage with the *MCPH2 *locus harboring the *WDR62 *gene. Three families (MCP26, MCP35, MCP67) were located in villages near Lahore in the Punjab province of Pakistan, while fourth family (MCP3) originates from Abbottabad in the Khyber Pakhtunkhwa province. The evaluation of the affected individuals of all families (Figure 1) revealed presence of microcephaly and mental retardation. In these patients, microcephaly was noticed at birth and their head circumferences were between -3 and -7 standard deviations. The affected individuals of these families exhibited similar developmental trajectory without any signs of facial dysmorphism, seizures and autism. Interestingly affected male individuals (IV-5, IV-6) of family MCP67 have abnormal sleeping habits. The computerized tomography (CT) scan of the affected individual IV-5 of family MCP67 revealed reduced volume of right cerebral hemisphere and prominent extra axial cerebrospinal (CSF) spaces with ill defined gryal and nuclei pattern (Figure 2). However no local area of brain attenuation and intracerebral blood was observed. Due to the non cooperative behavior and effectiveness of available sedative methods on this individual (IV-5) detailed magnetic resonance imaging (MRI) scan could not be performed.

**Figure 3:**
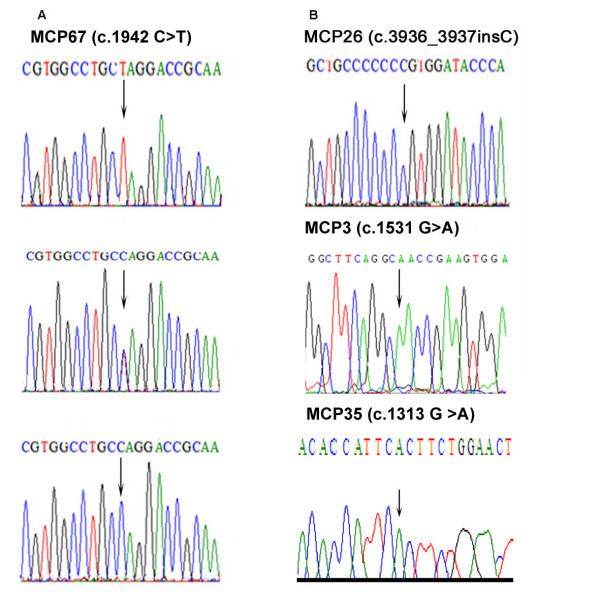
**Sequence chromatograms of four probands with *WDR62 *mutations**. **A) **Sequence chromatogram of family MCP67 with novel WDR62 mutation. The upper panel represents the nucleotide sequence in an affected individual, the middle panel in the heterozygous carrier and the lower panel in the unaffected individual. **B) **Sequence chromatogram of families MCP3, MCP26 and MCP35 with known WDR62 mutations. Arrows indicate the site of mutation.

DNA sequencing analysis of the entire coding region of *WDR62 *gene identified two truncating mutations (of which one novel) and two previously reported missense mutations (Figure 3). The novel mutation in family MCP67 is a single base pair transversion in exon 15 of the gene (c.1942 C>T) resulting in a premature stop codon (p.Q648X). In family MCP26 an already reported homozygous single base pair insertion (c.3936_3937insC) in exon 30 was observed that changed the reading frame, creating a premature stop codon 50 base pair downstream (p.Val1314ArgfsX18) (Figure 3). Additionally two missense mutations were identified in exon 10 (c.1313G>A; p.Arg438His) and 11 (c.1531G>A; p.Asp511Asn) in families MCP35 and MCP3, respectively. These mutations were homozygous in affected individuals and found heterozygous in obligate carriers and were not found in 100 control individuals of Pakistani origin.

## Discussion

To date 18 mutations have been reported in *WDR62 *gene (Table 1) which are associated predominately with primary microcephaly but additional features like pachygyria with cortical thickening and hypoplasia of the corpus callosum, schizencephaly and polymicrogyria were also found in few patients [1-4]. The current study describes identification of two protein truncating and two missense mutations in *WDR62 *gene in four consanguineous Pakistani families originating from Punjab and Khyber Pakhtunkhwa province.

**Table 1:** Pathogenic sequence variants in *WDR62 *gene reported to date in families mapped to *MCPH2*

S.No	Nucleotide change	Amino acid change	Location	Ethnic group	Ref paper
1	193 G>A	Val65Met	Exon 2	Arab	[2,3]

2	363delT	Asp112MetfsX5	Exon 4	Mexican	[3]

3	535_536insA	Met179fsX21	Exon 5	Indian	[4]

4	671 G>C	Trp224Ser	Exon 6	-	[1]

5	c.900C>A	Cys300X	Exon 8	Indian	[4]

6	1043+1 G>A	Ser348RfsX63	Intron 8	Turkish	[3]

7	1313 G>A	Arg438His	Exon 10	Pakistani	[2], This study

8	1408C>T	Gln470X	Exon 11	-	[1]

9	1531 G>A	Asp511Asn	Exon 11	Pakistani	[2], This study

10	1576 G>T	Glu526X	Exon 12	-	[1]

11	1576 G>A	Glu526Lys	Exon 12	-	[1]

12	1942 C>T	Gln648X	Exon 15	Pakistani	This study

13	2867+4_c2867+7delGGTG	Ser956CysfsX38	Intron 23	Turkish	[3]

14	3232 G>A	Ala1078Thr	Exon 27	Pakistani	[2]

15	3839_3855delGCCAAGAGCCTGCCCTG	Gly1280AlafsX21	Exon 30	Turkish	[1,3]

16	3936dupC/3936_3937incC	Val1314ArgfsX18/Val1314GlyfsX17	Exon 30	Caucasian, Turkish, pakistani	[2,3] This study

17	4205delTGCC	Val1402GlyfsX12	Exon 31	Eastern Turkey	[1]

18	4241dupT	Leu1414LeufsX41	Exon 31	Pakistani	[2]

The p.Val1314ArgfsX18 truncating mutation identified in family MCP26 had already been reported in a Turkish family [3], as well as in affected members of Caucasian family [2] with thickened cerebral cortex and simplified gryal pattern leading to severe microcephaly. The affected individuals of family MCP26 also have severe clinical features, but unfortunately CT and MRI scan were not available to make better comparison. In family MCP67, harboring the truncating mutation p.Q648X, the affected subjects also have severe clinical course and ill-defined gyral pattern on brain imaging, in line with the hypothesis that truncating mutations in WDR62 gene are associated with severe microcephaly and associated brain malformations [2]. Indeed, truncating mutations may lead to nonsense mediated decay which results in complete loss of Wdr62 protein, causing the absence of Wdr62 at the spindle poles of the dividing cells. However the affected individuals of families MCP3 and MCP35, carrying known missense mutations, showed milder symptoms and simplified gyral patterns (Data not shown).

Cerebral cortex, the most complex region of mammalian nervous system is formed by the orderly generation of postmitotic neurons during neurogenesis through proliferation and differentiation of neural progenitors at the ventricular and subventricular zones that line the cerebral cavity [12,13]. Initially neural progenitor cells divide symmetrically to generate a pool of neural precursors for lateral expansion of neocortex. Subsequently asymmetric neurogenic division perpendicular to their apical-basal axis (horizontal cleavage) renews the precursor cell along with generation of post mitotic neuron [14]. The switch of mammalian neuroepithelium (NE) cells from symmetric, proliferative to asymmetric, neurogenic divisions has been proposed to involve a change in cleavage plane orientation hence depends upon spindle pole positioning [14].

Wdr62 is a spindle pole protein expressed in neuronal precursor cells undergoing mitosis in the mammalian embryonic neuroepithelium [2-4] and hence vital for positioning the cleavage furrow for symmetric proliferative division to increase the number of neuronal precursor cells [2]. Therefore, mutated wdr62 may results in depletion of progenitor pool affecting the proliferation rate and thus produce reduced neuron numbers causing primary microcephaly. Nicholas et al. [2] furthermore hypothesized that *WDR62 *is also involved in neuronal migration and mutated wdr62 may cause cortical lamination defect along with impairing centrosomal function.

## Conclusion

This study supports the notion that *WDR62 *mutations results in brain malformations in addition to primary microcephaly irrespective of family origin and ethnicity. Furthermore it also indicates that beside *ASPM*, *WDR62 *gene is a relevant contributor for autosomal recessive primary microcephaly in Pakistan, being responsible for about 4% cases.

## Competing interests

The authors declare that they have no competing interests.

## Authors' contributions

MJH, SB, SM and AM identified, recruited and clinically evaluated the subjects. RK, MJH and BK did the DNA extraction, genotyping and homozygosity mapping. RK, AM and SB performed sequencing of *WDR62 *gene in four families and control samples. In addition to manuscript writing the study was designed and supervised by WA and MA. All authors have read and approved the final manuscript.

## Pre-publication history

The pre-publication history for this paper can be accessed here:

http://www.biomedcentral.com/1471-2377/11/119/prepub
